# Inappropriateness of health care in Canada: a systematic review protocol

**DOI:** 10.1186/s13643-019-0948-1

**Published:** 2019-02-11

**Authors:** Janet E. Squires, Ian D. Graham, Doris Grinspun, John Lavis, France Légaré, Robert Bell, Stephen Bornstein, Susan E. Brien, Mark Dobrow, Megan Greenough, Carole A. Estabrooks, Michael Hillmer, Tanya Horsley, Alan Katz, Christina Krause, Wendy Levinson, Adrian Levy, Michelina Mancuso, Alies Maybee, Steve Morgan, Letitia Nadalin Penno, Andrew Neuner, Tamara Rader, Janet Roberts, Gary Teare, Joshua Tepper, Amanda Vandyk, Denise Widmeyer, Michael Wilson, Jeremy M. Grimshaw

**Affiliations:** 1 Clinical Epidemiology Program, Ottawa Hospital Research Institute, 501 Smyth Road, P.O. Box 201-B, Ottawa, Ontario K1H 8L6 Canada; 2 Faculty of Health Sciences, School of Nursing, University of Ottawa, Ottawa, Canada; 3 Faculty of Medicine, School of Epidemiology and Public Health, University of Ottawa, Ottawa, Canada; 4 Registered Nurses’ Association of Ontario, Toronto, Canada; 5 Department of Health Research Methods, Evidence, and Impact, McMaster University, Hamilton, Canada; 6 Population Health and Practice Changing Research, Research Centre of the CHU de Québec, Québec City, Canada; 7 Department of Family Medicine and Emergency Medicine, Université Laval, Québec City, Canada; 8 Health and Social Services Systems, Knowledge Translation and Implementation Core, Quebec SPOR Support Unit, Montréal, Canada; 9 Department of Surgery, University of Toronto, Toronto, Canada; 10 Newfoundland & Labrador Centre for Applied Health Research, Memorial University, St. John’s, Canada; 11 Department of Political Science and Faculty of Medicine, Memorial University, St. John’s, Canada; 12 Public Reports, Health Quality Ontario, Toronto, Canada; 13 Institute of Health Policy, Management and Evaluation, Dalla Lana School of Public Health, University of Toronto, Toronto, Canada; 14 Faculty of Nursing, University of Alberta, Edmonton, Canada; 15 Information Management, Data, and Analytics, Health System Information Management Division, Ontario Ministry of Health and Long-Term Care, Toronto, Canada; 16 Royal College of Physicians and Surgeons of Canada, Ottawa, Canada; 17 Manitoba Centre for Health Policy, Department of Community Health Sciences and Family Medicine, University of Manitoba, Winnipeg, Canada; 18 British Columbia Patient Safety & Quality Council, Vancouver, Canada; 19 Institute of Health Policy, Management, and Evaluation, Department of Medicine, University of Toronto, Toronto, Canada; 20 Department of Community Health and Epidemiology, Dalhousie University, Halifax, Canada; 21 New Brunswick Health Council, Moncton, Canada; 22 Patients Advisors Network, Toronto, Canada; 23 Faculty of Medicine, School of Population and Public Health, University of British Columbia, Vancouver, Canada; 24 Canadore College, North Bay, Canada; 25 Health Quality Council of Alberta, Calgary, Canada; 26 Canadian Agency for Drugs and Technologies in Health, Ottawa, Canada; 27 Patient Collaborator, Toronto, Canada; 28 Alberta Health Services - Alberta Cancer Prevention Legacy Fund, Population, Public and Indigenous Health, Calgary, Canada; 29 Department of Family and Community Medicine, Institute of Health Policy, Management and Evaluation, University of Toronto, Toronto, Canada; 30 Faculty of Health Sciences, School of Nursing, University of Ottawa, Ottawa, Canada; 31 Manitoba Institute for Patient Safety, Winnipeg, Canada; 32 Clinical Epidemiology Program, Ottawa Hospital Research Institute, Department of Medicine, University of Ottawa, Ottawa, Canada

**Keywords:** Quality of health care, Health services research, Delivery of health care

## Abstract

**Background:**

There is increasing recognition in Canada and globally that a substantial proportion of health care delivered is inappropriate as evidenced by (1) harmful and/or ineffective practices being overused, (2) effective clinical practices being underused, and (3) other clinical practices being misused. Inappropriate health care leads to negative patient experiences, poor health outcomes, and inefficient use of scarce health care resources. The purpose of this study is to conduct a systematic review of inappropriate health care in Canada. Our specific objectives are to (1) systematically search and critically review published and grey literature for studies on inappropriate health care in Canada; (2) estimate the nature and magnitude of inappropriate health care in Canada and its provincial and territorial jurisdictions.

**Methods:**

We will include all quantitative study designs reporting objective or subjective measurements of inappropriate health care in Canada over the last 10 years. We will search the following online databases: MEDLINE, Cochrane Central Register of Controlled Trials, EconLit, and ISI-Web of Knowledge, which contains Web of Science Core Collection-Citation Indexes, Science Citation Index Expanded, Conference Proceedings Citation Index-Science, and Conference Proceedings Citation Index-Social Science & Humanities. We will also search grey literature sources to identify provincial and national audits of inappropriate health care. Two authors will independently screen, assess data quality, and extract data for synthesis. Study findings will be synthesized narratively. We will organize our data into three care categorizations: preventive care, acute care, and chronic care. We will provide a compendium of inappropriate health care for each care category for Canada and each Canadian province and territory, where sufficient data exists, by calculating (1) overall medians of underuse, overuse, and misuse of clinical practices and (2) the range of medians of underuse, overuse, and misuse for each clinical practice investigated.

**Discussion:**

This review will result in the first-ever evidence-based compendium of inappropriate health care in Canada. We will also develop detailed reports of inappropriate health care for each Canadian province and territory.

**Systematic review registration:**

PROSPERO CRD42018093495

**Electronic supplementary material:**

The online version of this article (10.1186/s13643-019-0948-1) contains supplementary material, which is available to authorized users.

## Background

As health systems in Canada and around the world struggle with sustainability [1], there is increasing recognition that a substantial proportion of the health care delivered is clearly inappropriate as evidenced by (1) harmful and/or ineffective clinical practices being overused, (2) effective clinical practices being underused, and (3) other clinical practices being misused [2–10]. Several studies have highlighted the negative effects that inappropriate health care may have on patients. In a study by Baker and colleagues [11], 20 hospitals were randomly chosen across 5 provinces in Canada (British Columbia, Alberta, Ontario, Quebec, and Nova Scotia) to identify adverse events and their preventability: an adverse event being defined as an unintended injury or complication, resulting in disability, death, or a prolonged hospital stay [11]. The methods of this study were based on several global studies from Australia [12], the UK [13], New Zealand [14–16], Denmark [17], and the USA [18–20]. A screening process was completed in 4164 hospital admissions, and physician reviewers identified a total of 1133 injuries or complications in 858 charts. In 401 of those charts (46.7%), the adverse events resulted in death, disability at the time of discharge, or prolonged hospital stay [11]. Overall, 7.5% of patients experienced one or more adverse event and 36.9% of those patients experienced highly preventable adverse events [11]. In response, there is widespread professional and policy interest in reducing such inappropriate health care in Canada. For example, in 2014 Choosing Wisely Canada (CWC), a campaign designed to help clinicians and patients engage in conversations about unnecessary tests/treatments, and make optimal choices to ensure high-quality appropriate health care, was established [21]. CWC, modeled after the US Choosing Wisely® campaign, is physician-led in partnership with the Canadian Medical Association and is now endorsed by all provincial/territorial medical associations in Canada [21].

Inappropriate health care leads to negative patient experiences [22], poor health outcomes [23, 24], and inefficient use of scarce health care resources [25]. Summaries of inappropriate health care, each revealing astonishingly high levels, exist for the USA [2, 9], the UK [4], and Australia [5]; no summary exists for Canada. In the USA, performance in preventive, acute, and chronic care was evaluated; care was found to be inappropriate (i.e., overused or underused) in all three care areas, with 30% of patients receiving inappropriate acute care services, 40% receiving inappropriate chronic care services, and 50% receiving inappropriate preventive care services [2]. A second more recent review in the USA focused only on overuse, one dimension of inappropriate care; overuse rates were substantial, ranging from a high of 89% for antibiotic use in upper respiratory tract infections to a low of 1.4% for coronary artery bypass graft [9]. In the UK, performance in primary care was evaluated and care was found to be inappropriate more often than not, ranging from a high of 97% inappropriate care for diabetic foot examination to a low of 51% for diabetic fundal examinations [4]. In Australia, performance in primary care was also evaluated; patients received inappropriate care in 43% of encounters, ranging from a high of 87% inappropriate care for alcohol dependence to a low of 10% for coronary artery disease [5]. These reviews have provided much needed additions to the field of health care quality by confirming that inappropriate health care is a serious and widespread problem, and one to which no health sector is immune. These reviews also laid the foundation for several highly successful quality improvement initiatives, e.g., the *100,000 Lives* and *Protecting 5 Million Lives from Harm* campaigns in the USA and Safer Healthcare Now! in Canada, which led to substantial reductions in inappropriate health care resulting in large numbers of saved lives [26, 27].

While no comparable comprehensive synthesis of inappropriate health care exists in Canada, it is reasonable to assume that similar high levels of inappropriate care exist in Canada. However, we do not know which types of care are inappropriately delivered; whether they are overused, underused, or misused; or how this inappropriate use may vary by province/territory, and the jurisdictional unit in Canada where most practice optimization efforts take place [2]. As a result, it is currently difficult to make informed judgments about which clinical practices to prioritize and where to focus quality improvement efforts in Canada. This task is further complicated by the fact that Canada does not presently have a mandatory comprehensive national quality tracking system. While the Canadian Institute for Health Information (CIHI), an independent organization that provides information on Canada’s health system and the health of Canadians, houses multiple pan-Canadian databases spanning different health sectors, it does not collect information on all clinical practices and contributions to their databases vary greatly by jurisdiction and health sector. Thus, in the absence of a mandatory and more comprehensive national quality tracking system, a systematic summary of published and grey literature is the best way to provide an overview of inappropriate health care in Canada [7].

## Defining inappropriate health care

Inappropriate health care is a core component of health care quality, which is a multidimensional concept comprised of (1) structure (organizational factors that define the health system, includes physical and staff characteristics); (2) care processes (interactions between users, includes clinical care and interpersonal care); and (3) outcomes (consequences of care) [28, 29]. In our review, we will focus on inappropriate health care (which are care processes), because this is the dimension of health care quality that is associated with the most waste in health care [2].

A scoping review [30] of health care *appropriateness* revealed two dimensions to the concept: (1) appropriate health care and (2) inappropriate health care. Both dimensions were consistently defined across studies: appropriate health care in terms of positive effects for patients and inappropriate health care in terms of negative effects for patients [30–34]. This notion of positive and negative effects was explicitly defined by researchers at the RAND Corporation (23) with the introduction of risks and benefits. According to RAND, a clinical practice was considered to be *inappropriate* when the “risks exceeded the expected benefit” [34] (p. 669). This definition applies to an “average patient” presenting to an “average physician” [30, 35]. Building on the RAND definition of inappropriate health care, several conceptual frameworks emerged that shed further light on what constitutes inappropriate health care. In 1996, Lavis [6] published a framework proposing that health care could be inappropriate along two dimensions: (1) the clinical practice (e.g., administration of a drug) and (2) the setting where the service is delivered (e.g., hospital). In this framework, an inappropriate clinical practice is one that is “not expected to benefit the patient or, in the more extreme case, may harm the patient” [6] (p. 326). In a second framework, Sharpe and Faden [32] furthered our understanding of inappropriate care by asserting that it includes both under- and overuse of clinical practices [32]. A third framework, from the US National Roundtable on Health Care Quality [36], used a tripartite classification to describe inappropriate health care: (1) underuse, (2) overuse, and (3) misuse. *Underuse* refers to a failure to provide a clinical practice when patient benefits clearly outweigh the risks (e.g., missing a childhood immunization for measles) [36]. *Overuse* occurs when a clinical practice is provided under circumstances in which its potential for harm exceeds the possible benefit (e.g., prescribing an antibiotic for a viral infection for which antibiotics are ineffective) [36]. *Misuse* occurs when an appropriate practice is selected but a preventable complication occurs resulting in the patient not receiving the full potential benefit of the practice (e.g., a patient suffers a rash after receiving penicillin for a strep throat (which is appropriate) despite having a known allergy to that antibiotic) [36]. Drawing on each of these conceptualizations, we define “inappropriate health care” for this review as inappropriate clinical practices that may harm the patient, or when health care risks exceed the benefits. We will use the Institute of Medicine’s [36] tripartite classification above of inappropriate clinical practices: (1) underuse, (2) overuse, and (3) misuse.

The *purpose* of the study described in this protocol is to conduct a systematic review of inappropriate health care in Canada to inform urgently needed quality improvement programs. This review will serve as a foundation of evidence-based knowledge for future progress in quality improvement provincially and nationally. Our *specific objectives* are to (1) systematically search and critically review published and grey literature for studies on inappropriate health care in Canada and (2) estimate the nature and magnitude of inappropriate health care in Canada and its provincial and territorial jurisdictions.

## Methods

### Protocol and registration

This protocol is reported following the Preferred Reporting Items for Systematic Reviews and Meta-Analyses Protocols (PRISMA-P checklist) [37]. The PRISMA-P checklist is included as an additional file [see Additional file 1]. This systematic review will be conducted in accordance with Cochrane’s principles for systematic reviews [38], with adaptations for observational study designs which are frequently used in studies of inappropriate health care. This protocol is registered with PROSPERO no. CRD42018093495. Any amendments to this study protocol will be documented and filed with PROSPERO.

### Eligibility criteria

We will include all quantitative study designs (e.g., randomized and quasi-randomized controlled trials, cohort, case-control, cross-sectional) reporting objective measurements (e.g., observation of practice, chart audits, administrative data) of inappropriate health care in Canada. We will also include subjective patient-reported measurements of inappropriate health care given some care processes are not routinely recorded in administrative data or patients’ charts (e.g., counseling). We define inappropriate health care as *inappropriate clinical practices*. In this review, an *inappropriate clinical practice* is one that does not conform to clinical practice recommendations that are based on strong research evidence [2, 4, 5, 32] (e.g., use of antibiotics in viral illnesses). We define *strong research evidence* as synthesized evidence from provincial/national evidence-based clinical practice guidelines, provincially/nationally developed quality indicators, or systematic reviews/meta-analyses. All types of inappropriate clinical practices will be eligible: underuse (i.e., failure to provide a clinical practice when patient benefits clearly outweigh the risks), overuse (providing a clinical practice when its potential for harm exceeds the possible benefit), and misuse (an appropriate clinical practice was selected but a preventable complication occurs, and as a result, the patient does not receive the full potential benefit of the practice) [36]. All clinical practices (e.g., clinical problem identification/screening, diagnosis, assessment, intervention/treatment or follow-up) undertaken by any healthcare professional (e.g., physician, nurse) in a Canadian health care setting will be eligible. In line with previous reviews of inappropriate health care from other countries [2, 4, 5, 9], we will limit inclusion to studies that report on large or diverse populations, such as the entire nation, a province/territory, a city, or multiple (> 1) centers. No restrictions based on language or practice funding (e.g., public, private) will be applied.

### Search strategy

Co-author (TR), an experienced information scientist, designed a sensitive search strategy informed by our scoping review [39] to retrieve studies from electronic bibliographic databases and grey literature sources. The search strategy was peer reviewed by an external librarian using the PRESS EBC Checklist, an evidence-based checklist for the peer review of electronic search strategies [40]. We limited our searching to Canadian studies from the last 10 years. See Table 1 for a search strategy for MEDLINE, which was translated for use in the other relevant electronic databases, including Cochrane Central Register of Controlled Trials, EconLit via ProQuest, and ISI-Web of Knowledge, which contains Web of Science Core Collection-Citation Indexes, Science Citation Index Expanded, Conference Proceedings Citation Index-Science, and Conference Proceedings Citation Index-Social Science & Humanities. We will also search grey literature sources to identify provincial and national audits of inappropriate health care. This will include contacting and searching for quality reports on the websites of all provincial/territorial Ministries of Health, provincial health care quality organizations (all of whom are represented on our team), and provincial (e.g., Institute for Clinical Evaluative Sciences) and national (e.g., Canadian Institute for Health Information) administrative data facilities. Identification and access to these grey literature reports will be facilitated by our national team. Additional sources of data will be identified by reviewing reference lists of relevant publications/reports; contacting authors of relevant publications/reports to clarify published and/or seek unpublished information; and contacting Canadian researchers and knowledge users, internal and external to our team, who possess expertise in inappropriate health care.

**Table 1:** Search strategy—Ovid MEDLINE

**#**	Controlled vocabulary (MeSH) and text word search terms
1	Health services research/
2	Health care reform/
3	“Health services needs and demand”/
4	Comparative effectiveness research/
5	Inappropriate Prescribing/
6	Quality Assurance, Health Care/
7	guidelines as topic/ or practice guidelines as topic/
8	“Quality of Health Care”/
9	(quality adj2 indicat*).tw.
10	Delivery of Health Care/
11	or/1-10
12	Guideline Adherence/
13	Critical Pathways/
14	exp Decision Theory/ or Decision Trees/ or exp Decision Support Techniques/
15	((clinical or diagnostic or practice or practice or physician) adj3 decision* adj3 (rule or tool or tools or pathway* or support* or model or models or system or systems)).ti,ab.
16	((CDST or CDSTs) and decision).ti,ab.
17	((pathway? or protocol? or algorithm?) adj2 (clinical or treatment? or diagnos* or management or infection? or infectious? or antibiotic?)).ti,ab.
18	critical pathway*.ti.
19	inappropriate.ti,ab,kf.
20	(quality adj2 (improv$ or manag$ or care or healthcare)).ti,ab.
21	((inappropriate or appropriate or overuse or overused or over-used or over-use) adj2 (care or approach or health care or healthcare or treat* or therap*)).ti,ab.
22	or/12-21
23	(canadian* or canada* or british columbia* or alberta* or saskatchewan* or manitoba* or ontario* or quebec* or new brunswick* or prince edward island* or nova scotia* or labrador* or newfoundland* or nunavut* or northwest territor* or yukon* or toronto* or montreal* or vancouver* or ottawa* or calgary* or edmonton* or winnipeg* or first nation* or metis).jw,ti,ab,hw,ot,kf.
24	((indigenous or aborig*) adj2 (people or person*)).ti,ab,kf.
25	exp Canada/
26	Or/23-25
27	11 and 22 and 26
28	limit 23 to yr=“2007 - 2019”

### Data collection and analysis

#### Identifying relevant literature

Screening will be undertaken using the Distiller Systematic Review Program [41], an Internet-based software program that facilitates collaboration among reviewers during the study selection and data extraction processes. Two team members independently, using a two-step process, will assess the results of the published and grey literature searches. In level 1, all records will be assessed against three screening questions which we piloted on a sample of 287 abstracts from the MEDLINE search: (1) Was a quantitative study design used? (2) Is there measurement of inappropriate health care (or appropriate health care from which inappropriate health care can be derived) in a Canadian health care setting? and (3) Does the study report on a large or diverse population or on multiple centers? All potentially relevant records, as well as those that do not contain enough information to determine eligibility, will be retained. In level 2 screening, the full text of all retained records will be obtained and assessed for inclusion against the same screening questions. All discrepancies on whether a study meets the inclusion criteria will be resolved by consensus and, where necessary, by consulting a third senior team member. Reasons for exclusion will be documented.

#### Data extraction

Two team members will independently extract data from all included studies using a standardized data extraction form into the Distiller Systematic Review Program [41]; one team member will extract data which will be verified for accuracy by a second team member. Data extracted will include **(**1) *study identification*—authors, publication year, province/territory, language, publication status, funding, study design, data collection dates, and care category (preventive, acute, chronic); (2) *participant characteristics*—sample description (of both the patients receiving the care and the health care professionals delivering the care) including their gender/sex, age, education, and sample size, and for the health care professionals—role (e.g., physician, nurse) and experience; (3) *clinical issue*—descriptions of the clinical practice and funding source (e.g., retinopathy screening in diabetes mellitus, publicly funded), recommended service and evidence source (e.g., type 1 diabetes mellitus—screen for retinopathy every year, Canadian Diabetes Association Clinical Practice Guideline [42]); and health care setting (e.g., primary care clinic); and (4) *outcomes*—data source (e.g., administrative database) and details of the inappropriate health care—e.g., percentage of patients who experienced underuse, overuse, or misuse of the clinical practice. In studies where only appropriate health care is reported, where possible, we will extract data to extrapolate inappropriate health care. In longitudinal studies, we will focus our analyses on the last reported time point; in any experimental studies, we will focus on baseline measurements for trials with baseline data and on post intervention control group data in all other trials. Where necessary, we will obtain additional data through communications with original study authors. We have successfully pilot-tested our data extraction form on 5 published studies [43–47]; prior to commencing data extraction in the review, we will pilot, and refine if necessary, our data extraction form on a sample of grey literature. Disagreements in data extraction will be resolved by consensus and by consulting a third senior team member if needed.

#### Methodological quality

Strength or quality of evidence determines the level of confidence authors place in any estimates derived from the study [48]. Therefore, two team members will independently conduct a methodological quality assessment of each included published study and grey literature report. Disagreements will be resolved through consensus and, if necessary, by consulting a third senior team member. Most studies on inappropriate health care, irrespective of whether it is a published study or in the grey literature, use cross-sectional designs (e.g., surveys, chart audits); we will assess these published studies/grey literature using the *Quality Assessment and Validity Tool for Cross-Sectional Studies*. This is a valid and reliable tool [49–55] developed based on medical literature [56, 57] and has been used in multiple systematic reviews of cross-sectional data (e.g., [49–55]). The tool uses 12 items to assess studies in three methodological domains: (1) sampling, (2) measurement, and (3) statistical analysis. Areas assessed under *sampling* include probability sampling, representativeness of the sample, sample size, number of sites, use of matching, and survey response rate. Areas assessed under *measurement* include main outcome measurement and its reliability and validity. Areas assessed under *statistical analysis* include statistical tests, *p* values, confidence intervals, and missing data. An overall quality score between 0 and 1 is derived using total points scored/total points possible. Using this overall score, each study/report will be classified as of < 0.5 weak, 0.50–0.74 moderate, or 0.75–1 strong methodological quality [50]. Other reliable and valid assessment tools will be used for published studies/grey literature that use other study designs as needed, e.g., the Revised Cochrane Risk of Bias Tool for Randomized Controlled Trials (RoB 2) [58] and Cochrane Risk Of Bias In Non-randomized Studies of Interventions Tool [59].

#### Data synthesis

This systematic review will require a narrative synthesis. We will organize our data into three categorizations of care: (1) preventive, (2) acute, and (3) chronic, preparing tables on each care category for Canada and for each province/territory where the data exists. This care categorization was chosen because there are important differences in the way each of these types of care is delivered that could affect whether or not care is inappropriate [2]. Preventive care is often initiated routinely by the clinician rather than on an episodic basis by a patient. The patient, on the other hand, typically identifies the need for acute care, which is frequently delivered during a single encounter. Chronic care is more likely than acute care to be delivered by a clinician who has an ongoing relationship with the patient [2]. These care categories were also used in the seminal US review of inappropriate health care [2] which led to multiple successful quality improvement initiatives [26]. Each table will contain data on setting, clinical practices investigated, sample, recommended service and its evidence source, study methodological quality, and inappropriate health care findings. We will develop a summary of inappropriate health care for each care category for Canada and each province/territory by calculating overall medians and ranges for underuse, overuse, and misuse of clinical practices (e.g., underuse, 45% of Canadians did not receive recommended preventive care). For clinical practices with a large number of included studies, we will calculate the interquartile range. We will conduct a sensitivity analysis to compare our median estimates in methodologically weak studies to all studies. Where data exists, we will also conduct subgroup analyses to determine whether inappropriate health care medians differ by (1) the evidential basis of the recommended practice indicator (i.e., by evidence source—clinical practice guideline vs. quality indicator vs. systematic review/meta-analysis), (2) the use of different measures of the same indicator, and (3) sex (women compared to men).

### Review quality

We will use the *Preferred Reporting Items for Systematic Reviews and Meta-Analyses (PRISMA)* statement [60], a checklist of 27 items for transparent reporting of systematic reviews, in reporting our review. We will also use the AMSTAR 2 tool [61], a 16-item quality appraisal tool for systematic reviews, as a guide to ensure our review meets quality standards and to avert any possible deficiencies in the conduct and reporting of our review. We will assess and report on the quality of the evidence across all studies using the GRADE approach [62], which involves consideration of five categories: methodological quality (risk of bias), imprecision, inconsistency, indirectness, and publication bias. We will rate the evidence in one of four categories—high, moderate, low, or very low quality [62].

## Discussion

### Potential challenges

One potential challenge that we may face is locating and securing unpublished provincial/territorial audits of inappropriate health care. In anticipation of this challenge, we purposefully designed our team to include executive-level knowledge users and/or senior researchers involved in health care quality from all Canadian jurisdictions; they will facilitate identification and access to these audits. A second potential challenge is the likely heterogeneity between studies in how inappropriate care is defined and measured, which would limit our ability to statistically compare Canadian jurisdictions on the same clinical indicators. Therefore, our analysis is designed to summarize inappropriate health care in each Canadian jurisdiction rather than to statistically compare jurisdictions—this is also seen by our knowledge users as the most valuable and actionable type of analysis. We will also conduct a subgroup analysis if sufficient data is available to compare inappropriate care when different measures are used to capture the same indicator.

### Limitations

Limitations to our review methods include relying on the identification of “recommended” clinical practices provided by the authors of our included studies. It is not feasible to assess the quality of the evidence behind each included study’s identified recommended clinical practices. Therefore, in line with previous reviews on inappropriate health care from other countries [2, 4, 5, 9], we will rely on the study authors’ identification of a “recommended” practice and document the evidence source provided. To ensure rigor, we will take several steps beyond what was done in the previous reviews; for example, we will limit inclusion to studies that provide reference to strong research evidence for their recommended practices: provincially/nationally developed clinical practice guidelines or quality indicators, or systematic reviews/meta-analyses. A second potential limitation to our methods is that some instances of inappropriate health care that will be identified may no longer be a problem today for a specified jurisdiction. To limit the likelihood of this occurring, we will restrict our searching to data collected in the past 10 years; according to our knowledge users, it takes at least 10 years to see trends in practice on which they would consider acting. A third possible limitation relates to the health care quality field in general; we will be able to capture only instances of inappropriate health care that have been studied, and therefore, our final estimates may be conservative.

## Conclusion

This project will be the first-ever systematic assessment of inappropriate health care in Canada and its jurisdictions. We will be able to provide an urgently needed snapshot of inappropriate health care in Canada and to develop the first-ever evidence-based Canadian compendium of inappropriate health care. We will develop provincial and territorial jurisdictional reports summarizing both the nature and magnitude of inappropriate health care in each jurisdiction. These reports will serve to advance much needed quality improvement programs at the provincial, territorial, and national government’s levels, as well as support agencies dedicated to quality and patient safety. It will enable the prioritization of programs and benchmark progress for quality improvement activities focused on improving health care quality and patient outcomes in Canadian jurisdictions.

## Additional file

Additional file 1: PRISMA-P 2015 Checklist. (DOCX 37 kb)

## Abbreviations

CIHI: Canadian Institute for Health Information
CWC: Choosing Wisely Canada
PRESS EBC: Peer Review of Electronic Search Strategies Evidence-Based Checklist
PRISMA: Preferred Reporting Items for Systematic Reviews and Meta-Analyses
PRISMA-P: Preferred Reporting Items for Systematic Reviews and Meta-Analyses Protocols
